# Long-term follow-up of left ventricular systolic function and functional status following Bentall procedure

**DOI:** 10.1186/1749-8090-8-S1-O17

**Published:** 2013-09-11

**Authors:** O Djokic, P Otasevic, S Micovic, S Tomic, I Petrovic, P Dabic, P Milojevic, B Djukanovic

**Affiliations:** 1 Dedinje Cardiovascular Institute, Belgrade, Serbia

## Background

Data are scarce on long-term effects of Bentall procedure on left ventricular systolic function and functional status. Aim was to assess long-term effects of Bentall procedure on left ventricular (LV) systolic function and functional status.

## Methods

Study group consisted of 90 consecutive patients who were operated using elective Bentall procedure for the aneurysm of the ascending aorta and aortic valve disease from 1997 to 2003 in a single tertiary care center. Patients were followed for eight years for mortality, LV ejection fraction and volume indices, as well as functional capacity as assessed by NYHA class. Echocardiographic measurements were made according to the recommendations given by the American Society of Echocardiography.

## Results

Study group consisted of 71 male and 19 female patients, mean age 54+/-10 years. There were no operative deaths. Survival rate was 73.3% during eight-year follow-up (11 cardiac and 13 non-cardiac deaths). Echocardiography was performed before index procedure and after 96+/-9 months. Statistically significant improvement in the LV ejection fraction was noted at follow-up examinations as compared to preoperative values (49.4+/-10.2% vs 42.5+/-10.9%, respectively, p<0.0001). Similarly, statistically significant reduction in the LV end-systolic (36.4+/-8 ml vs 59.2+/-29.1 ml, respectively, p<0.0001) and end-diastolic volumes (70.7+/-18.1 ml vs 101.4+/-32.1 ml, respectively, p<0.0001) were observed. NYHA class improved from baseline during the follow-up (3.1+/-0.8 vs 1.7+/-1.1, respectively, p<0.0001). Univariate analysis identified ejection fraction on admission and the presence of postoperative complications as predictors of long-term LV ejection fraction.

## Conclusion

Bentall procedure significantly improves long-term LV systolic function and functional status.

